# Evaluation of a population mobility, mortality, and birth surveillance system in South Kivu, Democratic Republic of the Congo

**DOI:** 10.1111/disa.12370

**Published:** 2019-10-16

**Authors:** Prudence Jarrett, Frank J. Zadravecz, Jennifer O'Keefe, Marius Nshombo, Augustin Karume, Les Roberts

**Affiliations:** ^1^ Research Fellow at the Mailman School of Public Health Columbia University United States; ^2^ North Kivu Regional Coordinator at Rebuild Hope for Africa Democratic Republic of the Congo; ^3^ Technical Advisor at Rebuild Hope for Africa Democratic Republic of the Congo; ^4^ Professor of Forced Migration and Health at the Mailman School of Public Health Columbia University United States

**Keywords:** community‐based surveillance, Democratic Republic of the Congo, evaluation, mortality, South Kivu, survey

## Abstract

Prospective, community‐based surveillance systems for measuring birth, death, and population movement rates may have advantages over the ‘gold‐standard’ retrospective household survey in humanitarian contexts. A community‐based, monthly surveillance system was established in South Kivu, Democratic Republic of the Congo, in partnership with a local implementing partner and the national ministry of health. Data were collected on the occurrence of births, deaths, arrivals, and departures over the course of one year, and a retrospective survey was conducted at the end of the period to validate the information. Discrepancies between the two approaches were resolved by a third visit to the households with discordant records. The study found that the surveillance system was superior in terms of its specificity and sensitivity in measuring crude mortality and birth rates as compared to the survey, demonstrating the method's potential to measure accurately important population‐level health metrics in an insecure setting in a timely, community‐acceptable manner.

## Introduction

Health‐related events such as births, deaths, and population movement speak to the quality of life in a community, and act as quantifiable measures of health and well‐being. However, conditions in emergency and post‐conflict settings pose substantial obstacles to measuring population‐scale health developments. In the absence of reliable census or vital registration statistics, mortality is the primary measure of the scale of population health needs and is essential for programmatic decision‐making in the corresponding humanitarian relief operation, as well as for advocacy (Checchi and Roberts, 2005). Consequently, the accuracy and timeliness of mortality measurement, as well as other vital variables such as the birth rate, have major implications for the effectiveness of this response and the health of the affected population.

There are two main methods for measuring mortality in such environments, each of which has important advantages and potential drawbacks. The first, retrospective household surveys, are considered to be the *gold standard*. These surveys are conducted over a short period of time and entail visits to a sample of households to collect data on the occurrence of vital events over a defined preceding period (for instance, the past year). They do not require a known population denominator, a useful attribute in development and humanitarian environments that often is not known, and necessitate only a brief amount of technical input and supervision (the length of the survey). However, estimates of mortality and birth rates are calculated long after the fact when it is frequently too late to respond to evolving trends. There is also the possibility of recall bias given a long recall period, so respondents may not be able to remember if the event occurred precisely within the specified time frame. Furthermore, if the population perceives the survey activity as a chance to receive assistance, the survey team being perceived as an agency registering the population for distributions, for example, household composition and the occurrence of births and deaths may be falsely reported (Checchi and Roberts, 2005)

In contrast, the other major method is prospective surveillance in which households are visited on a regular basis (such as weekly or monthly), often by a monitor or health worker based in the community of the selected households. The regular and ongoing collection and reporting of births and deaths can provide early warning of increasing mortality in a sufficiently timely manner to inform the aid response (Fatusić et al., 2005). In addition, repeated visits to households theoretically accord a surveillance approach greater flexibility, as new questions can be added to the schedule in response to evolving trends or operational needs. What is more, they may reduce recall bias owing to the shorter recall period and the fact that as the surveillance becomes a routine activity, it is not associated with receiving benefits so may be less of an incentive to make false reports (Checchi and Roberts, 2005). Expert working groups in emergencies have called for such community‐based, prospective surveillance (Purdin et al., 2009), yet it has rarely been employed outside of camps (Caleo et al., 2012). It has the limitation of needing a robust population denominator to calculate vital event rates, and the additional labour for ongoing data collection, supervision, and funding over the longer period of surveillance.

There is little published evidence of the feasibility and effectiveness of prospective surveillance. Caleo et al (2012) implemented a community surveillance system in a rural population in the Central African Republic (CAR), involving weekly household visits over a 32‐week period. They were able to demonstrate high excess mortality in this population with good sensitivity (92.9 per cent) for capturing deaths through surveillance, as evaluated in a capture–recapture analysis. They had difficulty, though, in accurately monitoring in‐ and out‐migration from the households, essential for calculating the population denominator for birth and death rates. Caleo et al (2012) conducted a demographic census of the households at the beginning and end of the surveillance period, arriving at a smaller population denominator than estimated using the surveillance system; they emphasise that they would have underestimated the true mortality rate without taking this step.

In Malawi, an existing cadre of community health workers was trained to monitor births and deaths with a focus on child mortality, but they found that this underestimated rates as compared to a household survey (Amouzou et al., 2014). Similarly, a community‐based mortality surveillance system used by Medécins Sans Frontières (MSF) in several refugee camps in Chad also found an underestimate of deaths as compared to survey data (Bowden et al., 2012).

As such, the population mobility, mortality, and birth surveillance system was established with provincial and district health authorities in the Fizi Health Zone, South Kivu, eastern Democratic Republic of the Congo (DRC), to determine if the monthly collection of demographic and mobility data at the household level can assess accurately the true occurrence of births, deaths, and persons moving into and out of monitored households. Two decades of conflict in eastern DRC have increased the already substantial obstacles to measuring health events among its population. Violence and instability are ongoing in South Kivu, with continued activity by armed groups. A system of volunteer community health workers presently exists in many of the country's health zones, known as *Relais Communautaires*, or ReCos. They are inhabitants of the community in which they work, are chosen by the residents of that community, and are meant to distribute key messages as guided by health professionals, assist with campaigns, and procure information on births and deaths. They are unpaid—unless supplemented by non‐governmental organisations (NGOs)—often poorly supported, and only a tiny fraction report health statistics.

The United Nations (UN) estimated in 2013 that the crude birth rate for the DRC was 42.9 births per 1,000 people per year, whereas the crude death rate was estimated at 15.5 per 1,000 people per year (United Nations, 2013). The infant mortality rate is estimated at 109.4 per 1,000 live births (UNFP, 2011) and the under‐five mortality rate at 180 per 1,000 live births (United Nations Department of Economic and Social Affairs, Population Division, 2013). In South Kivu, however, mortality has been measured at between two and four times these UN national estimates (Roberts et al., 2003).

Yet, despite population movement as a result of the conflict and continuing attacks, no system is in place to measure adequately vital statistics at the community level. This is especially alarming owing to the disproportional impact of negative health events on internally displaced persons (IDPs) (Thomas and Thomas, 2004).

### Objectives

This study had the following four aims:

- to establish a community‐based surveillance system for measuring births, deaths, and population movement in rural eastern DRC;
- to evaluate the sensitivity, specificity, and positive predictive value (PPV) of the system through a household survey conducted after one year of surveillance;
- to explore the characteristics of the surveillance system in relation to the Centers for Disease Control and Prevention's (CDC) criteria of simplicity, flexibility, data quality, acceptability, representativeness, timeliness, and stability (CDC, 2001); and
- to assess the feasibility of using such methods to measure mortality in humanitarian settings.

## Methods

### Ethics

The Institutional Review Board of Columbia University, United States, and the ministry of health (MoH) in Fizi Territory, DRC, provided clearance for the local collection of data. Oral informed consent was acquired from participants for each phase of surveillance, evaluation, and re‐evaluation. The study was jointly funded by CDC and Rebuild Hope for Africa (RHA), the local implementing partner of Columbia University.

### Study design

The study consisted of three stages: (i) the establishment and collection of data for the community‐based surveillance system; (ii) a survey‐based evaluation of the surveillance system; and (iii) a final re‐evaluation to confirm discrepancies between the first two stages.

### Setting

The DRC is a low‐income country with a young population: 45 per cent of the people are under the age of 15 years. The total fertility rate is 5.9 per woman and there is a high maternal mortality ratio of 730 per 1,000 live births. Life expectancy at birth is approximately 52 years (World Health Organization, 2015).

The population mobility, mortality, and birth surveillance system resides under the jurisdiction of the chief medical officer (MCZ) of Fizi Territory's MoH, and within the evaluation arm of the South Kivu office of RHA. The surveillance system utilises MoH‐supported volunteers, ReCos, and RHA programme and field staff.

### Surveillance events

All events under surveillance were amassed at the household level. Within the system where polygamous households were common, a household was defined as all of the persons who eat their meals together. The data points collected were as follows:

- Household composition—age and sex of each household member.
- Births—all live births to members residing within the household at the time of delivery, occurring within the past month. The sex, location (at a facility or at home), and month of birth were recorded.
- Deaths—all deaths of members residing within the household at the time of death, occurring within the past month. The age and sex of the deceased, as well as the cause of death as understood by the interviewee, were recorded. Deaths in clinics and hospitals were considered to be household deaths.
- Arrivals—the sex, age, reason, and month of arrival were recorded.
- Departure—the sex, age, reason, and month of departure were recorded.
- An arrival or departure event is included in the system if it has a duration of at least two weeks. Hence, those who departed or stayed for less than two weeks did not contribute to the population denominator. The reason for the arrival or departure was recorded.

The above surveillance events were used to calculate the mid‐period population at risk, and then the crude mortality rate and the crude birth rate. The under‐five mortality rate was not calculated.

### Surveillance population and sampling

The surveillance population was defined by 34 clusters spread across the Fizi Health Zone's catchment area, corresponding to 34 health centres. Initially, 40 clusters were chosen using primary sampling proportional to population size from a list of health centres within the health zone. Insecurity prevented the inclusion of the selected village in five of the selected areas, and the logistic constraints of difficult terrain and road conditions were considered insurmountable in one village.

**Figure 1:**
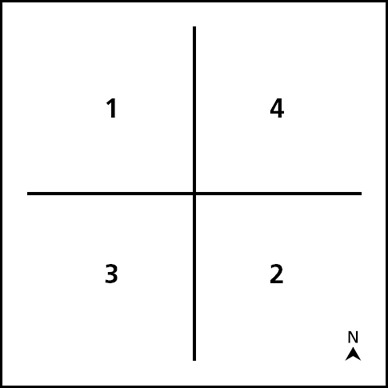
Village level survey quadrants for household sampling Source: authors.

Cluster‐level sampling was performed by dividing the village into north–south/east–west‐oriented quadrants and flipping a coin twice to choose the quadrant for sampling (see Figure 1). Houses within the selected quadrant were numbered and a random number was selected from a Congolese Franc bill for the random start within the quadrant. From this random start, 15 houses were then sampled in a northwest direction.

If homes were unoccupied, did not consent to participate, or were unavailable for survey, the next house was chosen for inclusion. If a quadrant was sampled and did not yield a sufficient number of homes for survey, the next numerical quadrant was chosen to complete the sampling (see Figure 1). The ReCos who was closest in proximity to the selected household was chosen as the monthly field monitor.

The sample size was based on an assumed crude mortality rate of 15.5 per 1,000 persons per year, and a crude birth rate of 35 per 1,000 persons per year. Using a 95 per cent confidence interval, and 80 per cent power to detect a doubling of the mortality rate between exposed (surveillance) and unexposed (non‐surveillance) households, a target sample size of 3,999 individuals was calculated. An assumption of six persons per household suggested that approximately 667 households were needed. A 2:1 ratio of unexposed to exposed persons resulted in sample‐size estimates calling for the evaluation of 223 surveillance households and 446 non‐surveillance households. Drawing on the available 34 clusters, the decision was taken to evaluate 15 surveillance houses and 30 non‐surveillance houses within the same cluster, yielding a potential 9,180 persons. This sample size offered sufficient power also to detect an increase in the crude birth rate of 50 per cent, assuming a rate of 35 births per 1,000 persons per year among the surveillance households.

Data collection was conducted in three stages described in detail below: monthly community‐based surveillance; a retrospective survey for comparison; and a re‐evaluation of discordant events between the surveillance and survey methods.

### Stage 1: surveillance

ReCo training occurred over four days in September 2011, and focused on how to define a household, gain oral consent, and collect and document the required data points in the notebook. Data were collected in French or Swahili. The tool was piloted during the training period and modified based on that experience. Collection commenced for the month of October 2011, and the same households were then visited on a monthly basis through October 2012. The recall period for the surveillance programme was defined as 1 November 2011 to 30 September 2012 (period length: 335 days). As a presidential election took place in November 2011, this was a convenient date for the start of the survey recall period.

At the end of each month, and following data collection by the ReCo, the RHA field supervisor personally visited each cluster to transcribe the data from the field books as obtained by the ReCos and to provide supervision. If discrepancies arose or questionable events appeared, the RHA fieldworker reviewed the occurrence with the ReCo and if necessary, revisited the household in question for clarification. This visit also provided an opportunity for one‐on‐one reinforcement of training, as the ReCo and supervisor examine the data and any questions or errors together.

The supervising fieldworker then entered the data into an electronic database in Microsoft Excel format and uploaded photocopies of the notebooks to a linked DropBox account. These two sources of data were then compared by a Columbia University external reviewer to verify accurate transcription of data, and to monitor the rates of events reported.

### Stage 2: survey‐based evaluation

Six ReCos who were already participating in data collection for the surveillance system were chosen for training in how to conduct the household survey. ReCos were not permitted to interview households within their own cluster and were not informed of the surveillance or non‐surveillance status of the household. The evaluation for the 34 surveillance system clusters took place between 29 August and 14 September 2012.

The evaluation utilised the initial sampling frame quadrants from the surveillance system. Events occurring within the 15 households that were monitored via the surveillance system were re‐evaluated in a household survey by an outside interviewer. In addition, households that were outside of surveillance monitoring were sought for comparative estimations of event rates. Thirty households that were not included in the surveillance system were chosen in a southwest direction from the southwestern‐most surveillance household, and were located in quadrant two (see Figure 1). If homes were unoccupied, did not have an eligible adult present, did not provide informed consent, or were unavailable for survey, the next house was selected for inclusion. Empty households were revisited at least once, although often on the same day. If there were not sufficient households within the quadrant, the next quadrant was taken and households were sampled to meet the requirement in a northwest direction.

Survey respondents had to be at least 18 years of age and give oral informed consent. When possible, the survey team requested the participation of a male or female head of household, but in their absence, a proxy who met eligibility requirements and permanently resided in the household was interviewed instead. Recalls of the occurrence of births, deaths, arrivals, and departures were requested for the period from the day of interview back to November 2011. The recall period for the evaluation was defined as the length of time between 1 November 2011 and the median day of the survey. For this survey, which happened between 29 August and 14 September 2012, the median day was 6 September, representing the end of the recall period for the evaluation (period length: 311 days).

### Stage 3: re‐evaluation

Two RHA staff interviewers performed a re‐evaluation from 5–16 December 2012 to investigate discrepancies in birth and death reporting between the surveillance system and the household evaluation survey. A discrepancy was defined as a birth or death event reported by one system but not by the other, without consideration of date or sex. The re‐evaluation was delayed because of security events in South Kivu Province. Total lists of discrepancies were produced to determine households that needed to be revisited. RHA interviewers then went to each household with a discrepancy and re‐interviewed the occupants to ascertain the appropriate resolution: true event or true non‐event. This procedure involved exploring the relationship of this person with the household, the timeline of other events before and after the death, their job, where they had been sleeping before the birth or death, and the symptoms and the course of the illness (for deaths). There was never an episode where resolution of discordance could not be achieved to the satisfaction of the staff.

### Analysis

Rates were calculated for both surveillance and survey‐based evaluation population data collection. These calculations only included data for months where both the surveillance and survey data were complete (October 2011 to August 2012) and were restricted to households that were accounted for at all three stages: the surveillance system; the survey; and the re‐evaluation. Survey‐based evaluation population data are reported separately for households assessed both within and without the surveillance system (see Table 1). A gold‐standard estimate was derived from triangulation of the three stages of the study. Births and deaths were considered to be the gold standard when: surveillance and survey‐based evaluation results matched; or, for those households with a discrepancy between surveillance and survey reports, the third‐stage re‐evaluation resolved the discrepancy. Box 1 provides formulae for rate calculations and definitions.

**Table 1:** Crude birth and death rates from surveillance and survey data before the re‐evaluation phase

Estimate	Midpoint population (n)	Births (n)	Crude birth rate (per 1,000 persons per year)	Deaths (n)	Crude mortality rate (per 1,000 persons per year)
Surveillance system	2,926	150	55.84	23	8.56
Evaluation (surveyed households)	2,730	177	76.11	56	24.08
Evaluation (non‐surveyed households)	7,003	425	71.23	195	32.68

**Source**: authors.

**Box 1. Definitions**

**Mid‐period population at risk**

Total population in sample + one‐half of the deaths in the sample during the recall period ‐ one‐half of the live births during the recall period.

**Crude mortality rate (per 1,000 persons per year)**

[Total deaths during recall period ÷ (mid‐period population at risk × duration of recall period)] × 1,000.

**Crude birth rate (per 1,000 persons per year)**

[Total live births during recall period ÷ (mid‐period population at risk × duration of recall period)] × 1,000.

**Sensitivity**

Number of concordant events (births or deaths) detected by the system (surveillance or evaluation) ÷ the total number of true events as determined by concordant reports during the December re‐evaluation (gold standard).

**Specificity**

Number of concordant non‐events (no births or deaths) picked up by the system (surveillance or evaluation) ÷ the total number of true non‐events determined by gold‐standard dataset.

**Positive predictive value**

Number of concordant events picked up by the system (surveillance or evaluation) ÷ the total number of events as detected by the system.

The unit of measurement for *sensitivity, specificity*, and *positive predictive value* is the household‐month in which an event occurred or did not occur.

**Source**: authors.

## Results

Originally, 510 households were included in surveillance with a population of 2,989 people, of which 51.6 per cent were female and 48.4 per cent were male. The surveillance population was slightly younger than for the DRC as a whole: 52 per cent of the population under 15 years of age.

Eighteen per cent of households (92 of 510) under surveillance were missed during the evaluation phase. Of these, 90 were on leave for an extended period of time and not due to return (37.0 per cent), one refused to answer the survey (1.1 per cent), and one household (1.1 per cent) had merged into another surveillance household over the period of the activity. A total of 1,420 households were reached in all three stages, therefore: 418 under surveillance households; and 1,002 non‐surveillance households. The 1,420 households interviewed comprised a total of 9,908 persons (surveillance: 2,790; non‐surveillance: 7,118), an average of 6.98 persons per household (surveillance: 6.67; non‐surveillance: 7.10).

Table 1 displays the surveillance and survey data in the absence of re‐evaluation triangulation. Initial surveillance data indicate 150 births and 23 deaths, with a midpoint population of 2,926 over the recall period. These numbers correspond to 55.84 live births and 8.56 deaths per 1,000 persons per year, with a range between the clusters of 13.29–145.27 live births and 0–30.27 deaths per 1,000 persons per year.

Among surveillance households, survey‐based evaluation data indicate 177 births and 56 deaths, with a midpoint population of 2,730 over the recall period. These numbers correspond to 76.11 live births and 24.08 deaths per 1,000 persons per year, with a range between the clusters of 25.19–155.92 live births and 0–77.23 deaths per 1,000 persons per year.

Among non‐surveillance households, survey‐based evaluation data indicate 425 births and 195 deaths, with a midpoint population of 7,003 over the recall period. These numbers correspond to 71.23 live births and 32.68 deaths per 1,000 persons per year, with a range between the clusters of 29.27–131.67 live births and 0–67.88 deaths per 1,000 persons per year.

Table 2 displays the results following the re‐evaluation. After investigation of the 214 event mismatches (161 birth mismatches and 53 death mismatches) between the initial surveillance and evaluation measurements, a gold‐standard estimate derived from surveillance–evaluation concordance and re‐evaluation triangulation indicated the occurrence of 129 births and 23 deaths over the recall period, corresponding to 51.60 live births (95 per cent confidence interval (CI) 42.7–60.5) and 9.20 deaths per 1,000 persons per year (95 per cent CI 5.4–12.9). In development of the gold standard, 130 of 161 birth (80.8 per cent) and 51 of 53 death (96.2 per cent) event discrepancies were resolved. Three clusters were removed from analysis (495 household‐months) and rate denominators (45 households). Two of these clusters were unable to be re‐evaluated and the third involved a ReCo who had not been monitoring households correctly over the recall period. A further 22 households were unable to be re‐evaluated and were removed from the analysis. In total, 31 birth (19.3 per cent) and two death (3.8 per cent) event discrepancies were unable to be resolved adequately.

**Table 2:** Crude birth and death rates from surveillance and survey data after the re‐evaluation phase

Error	Births (%)	Deaths (%)
Outside recall bounds	11 (17.2)	12 (31.6)
Not within household	40 (62.5)	18 (47.4)
Respondent false report	13 (20.3)	8 (21.1)

**Source**: authors.

Referencing the gold standard, the surveillance system correctly detected 120 of the 129 births (93.02 per cent) and 20 of the 23 deaths (86.96 per cent), with two false positives in each. Meanwhile, the survey‐based evaluation was shown to have correctly detected 85 of 123 births (69.11 per cent) and 15 of 21 deaths (71.43 per cent). Six households were excluded from the sensitivity and specificity analysis as the households in which these events occurred had been missed during the year‐end household survey. The initial survey‐based evaluation then produced 64 false positives within birth estimates and 38 false positives within death estimates. These were categorised in three ways: (i) outside recall bounds; (ii) not within household; or (iii) respondent false report. The distribution of false positives within the evaluation is described in Table 3. False positives labelled ‘outside recall bounds’ were events provided by interviewees that actually occurred outside of the designated recall period; false positives labelled ‘not within household’ were events provided by interviewees that occurred, but were not within the defined household; and false positives labelled ‘respondent false report’ were events either provided by interviewees intentionally to gain access to greater resources in the event of an intervention, or for any other undetermined reason.

**Table 3:** Distribution of false positives in survey‐based evaluation

Report	Sensitivity	Specificity	Positive predictive value
**Births**			
Surveillance	93.02	99.96	98.36
Evaluation	69.11	98.49	57.05
**Deaths**			
Surveillance	86.96	99.96	90.91
Evaluation	71.42	99.11	28.3

**Source**: authors.

In the initial evaluation, at least 23.2 per cent (41 of 177) of births involved women who arrived in a household, gave birth, and either left shortly afterwards or listed the reason for their arrival as care. Of these, 73.2 per cent (30 of 41) were not picked up in the initial surveillance. Likewise, 32.1 per cent (18 of 56) of deaths detected in the initial evaluation occurred concurrent with a group of arrivals. Of these, 66.7 per cent (12 of 18) did not show up in the initial surveillance.

### Sensitivity, specificity, and positive predictive value

Globally, the surveillance system is shown to have a sensitivity of 93.02 per cent and a specificity of 99.96 per cent with regard to the detection of births, and a sensitivity of 86.96 per cent and a specificity of 99.96 per cent with respect to the detection of deaths. The surveillance system for births and deaths has a positive predictive value of 98.36 per cent and 90.91 per cent, respectively (see Table 4).

**Table 4:** Summary of system performance

Estimate	Midpoint population (n)	Births (n)	Crude birth rate (per 1,000 persons per year)	95% CI	Deaths (n)	Crude mortality rate (per 1,000 persons per year)	95% CI
Gold standard	2,724	129	51.6	42.7–60.5	23	9.2	5.4–12.9
Surveillance update	2,724	120	48	39.4–56.6	20	8	4.5–11.5
Evaluation update	2,609	85	38.24	30.1–46.4	15	6.75	3.3–10.2

**Source**: authors.

The one‐time survey evaluation of events is shown to have a sensitivity of 69.11 per cent and a specificity of 98.49 per cent with regard to the detection of births, and a sensitivity of 71.42 per cent and a specificity of 99.11 per cent with respect to the detection of deaths. The survey for births and deaths has a positive predictive value of 57.05 per cent and 28.30 per cent, respectively.

Regarding community population movement, the surveillance system detected 1,868 arrival and 2,011 departure events at the household level over the evaluation period. Arrivals comprised 48 per cent of movement events, and departures 52 per cent. There was an estimated average arrival rate of 33 persons per 100 households per month and a departure rate of 36 persons per 100 households per month. Sensitivity and specificity of population movement events were not calculated, as a gold standard was not developed: surveillance and survey‐based evaluation discrepancies were not investigated because of security‐related time constraints during the month of re‐evaluation (December 2012).

## Discussion

This study contributes to the sparse published experience of prospective mortality surveillance in humanitarian settings and demonstrates the potential for a highly sensitive, specific, and feasible system of community‐based monitoring of births and deaths. The surveillance system was shown to outperform a traditional one‐year recall survey in both the correct detection of births (120 of 129; 93.02 per cent) and deaths (20 of 23; 86.96 per cent).

This is in contrast to studies from both Chad (Bowden et al., 2012) and Malawi (Amouzou et al., 2014). An existing cadre of community health workers in Malawi was trained to monitor births and deaths with a focus on child mortality, but found that this underestimated rates as compared to a household survey. Similarly, as mentioned earlier, a community‐based mortality surveillance system employed by MSF in several refugee camps in Chad also discovered an underestimate of deaths as compared to survey data. It may be that incentives to report deaths were different in those settings as compared to in Fizi Territory where no population‐based benefits or distributions were being provided.

However, the re‐evaluation stage of the study presented in this paper was not performed in the previous examples and was a crucial step in displaying the superiority of surveillance data in this case. Without investigating the discrepancies in recorded births and deaths that revealed the recall errors, definitional differences, and false reports present in the survey, it would have appeared that the surveillance system did indeed underestimate births and deaths in the same way as the two studies noted above, reiterating the already established potential for bias in retrospective surveys.

The magnitude of the discrepancies found here are highly unusual, though, and are probably the product of a unique combination of factors in this particular population at this particular moment in time. For instance, a large portion of the population of Fizi Territory had been in refugee camps in Tanzania during the preceding six years (ADEPAE and SVH, 2011), and those people had transitioned from full food rations and comprehensive education and healthcare services to little or no services back home, to the displeasure of many. This may have created unusual incentives to report their situation as aid worthy.

The surveillance system implemented in this study involved community monitors who were already known to their communities, a process of regular one‐on‐one field‐based supervision throughout the surveillance period, and a multi‐step process for checking data quality. These three features may also have contributed to the relatively better performance of the surveillance system in this case.

Despite the high sensitivity and specificity of this system, however, it is still underreporting births (‐3.6 live births per 1,000 persons per year) and deaths (‐1.2 deaths per 1,000 persons per year) as compared to the gold standard. As pointed out above, the evaluation of discrepancies between the surveillance system and survey resulted in 31 discrepant births and two discrepant deaths being removed from the analysis, making it likely that some of the events that truly occurred during the recall period were not counted, resulting in an underestimate of the birth and death rates.

Yet, many factors also affected whether an event was detected in the initial surveillance or survey‐based evaluation, such as the definition of a household, which is purposefully flexible as to be inclusive within the cultural definition of a household. Families often do not distinguish between the Western definitions of ‘extended’ and ‘immediate’ family, and visitors are common, frequently staying for longer than 30 days at a time. Although a household may be composed of many different buildings and individuals, eating meals together is a simple way of distinguishing related groups. The survey‐based evaluation counted each individual eating his or her meals at the house at the time of the interview as part of the household. Although the surveillance system was expected to employ the same definition, all four false positive cases among those verified (two erroneous births and two deaths) appeared to arise from the ReCo continuing to incorporate events that occurred in a family after they had left the residence under monitoring, such that they were no longer in the household. Failure to verify whether or not event person(s) stayed under the household roof on the night before the event occurred may have contributed to these inconsistencies.

The second most common reason for false birth and death reports was the inclusion of events that actually happened before the recall period. This is a phenomenon that has been witnessed in the region before (Bennouna et al., 2016).

Another reason for underreporting could be related to movement of the population for healthcare. This is extremely common, whether persons are receiving treatment for an illness or having a child. Women in particular will travel to deliver their baby in a health centre, and to be closer to extended family members for the process. Births in these circumstances often were missed by the surveillance system. The same is true of deaths that occurred among recently arrived groups. For many, the reason listed for arrival was mourning. But in several cases, this was a recall error. If the individual or group travelled to receive care and it was ultimately unsuccessful, the interviewee remembered the death as the defining event, as opposed to the initial aim of receiving care.

Furthermore, age at death may have been a factor strongly associated with deaths seen in the evaluation but not reported within the surveillance. When birth and child mortality rates are extremely high, as is the case in South Kivu, deaths in young children may not have the same implication as the death of an adult or older child. It is possible that because these events are not rare, and may affect members of a family differently, the surveillance system may be more likely to overlook them—underreporting of deaths among the very young has been previously reported in the country (Taylor et al., 1993). Indeed, because of this phenomenon, some such deaths may have been entirely missed by both the surveillance and survey‐based systems.

### System attributes: simplicity and flexibility

The system collects a small number of data points that do not require specialised interviewing techniques. As such, data collection can be done by monitors with a basic level of education and without intensive training. It is a relatively quick and simple procedure, therefore, to implement. Although the system is organised to require a minimal time commitment, it still necessitates continuous data collection and supervisory visits with consequences for monitor incentives and respondent fatigue (see below). Hence, the maintenance of the system is more involved than that for a one‐off survey. The system was not continued beyond one year, and the above constraints certainly call its sustainability into question.

The flexibility of the system is key to its use in fragile and remote contexts—when data collection is interrupted by insecurity or weather, the ReCo can delay the visit or follow up during the next month. The same can be done for supervisory visits. Clearly this will impact on the timeliness of any response to evolving trends, but it is preferable to missing the information entirely.

### Data quality

The system encompasses three steps at which the data can be checked. First, when the supervising fieldworker visits the ReCo each month, he/she checks the data and transcribes it into a compilation notebook, with the chance to feed back to the ReCo then and there and to ask him/her to clarify or complete the data on the next household visit. Second, when the supervisor enters the compiled data into the electronic database. Third, when the programme staff in the US review the electronic data.

The three‐point check system is intended to catch errors so that measures can be taken to reduce them, yet it is also possible that repeated transcription introduces mistakes. As telephone‐based systems for data collection in such contexts improve, this could be a way to mitigate this issue.

### Acceptability

By utilising the ReCos, the population mobility, mortality, and birth surveillance system is incorporating monitors who are already familiar to the community. Nonetheless, ReCos and fieldworkers underscored the presence of interview fatigue among surveillance households. Families become tired of answering the same questions month after month with no observable benefit to themselves or their household. If acceptability decreases, the quality of interviewee data may be compromised, as interviewees become less inclined to report events fully. When evaluating interviewers came to question the surveillance households, many were noticeably weary of responding about events. This may have been a motivation for interviewees to leave out information in order to speed up the process. In one case, the family under surveillance refused to be interviewed at all. Monitors themselves may also become fatigued as households become more reluctant, and thus less attentive to high‐quality data collection.

### Timeliness

Within the current system, the fieldworker is supposed to procure cluster data during the same week each month. In practice, logistical constraints frequently affect the time period in which data can be amassed, which may not be timely enough for events that require a quick response (an outbreak or a heightened number of casualties, mass arrivals, or an exodus). In this study, 8.7 per cent (N=354) of all events (N=4,053) detected in the initial monthly surveillance were outside of that specific month of monitoring owing to familial absence, a delay in data collection by the ReCo, or the discovery that a family omitted a previous event.

### System stability and movement

Fizi Territory in South Kivu poses unique challenges to surveillance system stability. Security within the catchment area is constantly changing with the fluid formation and dissolution of armed groups and the associated political climate. If the movement of persons within the area is restricted, surveillance system data collection can suffer at multiple stages. Likewise, the poor quality of roads in the area and the presence of a nine month‐long rainy season present considerable problems for travel. The stability of the system may be affected, too, if at any level a party, because of choice or logistical constraints, opts not to remain involved in the project. Despite the logistical constraints posed by a post‐ and current‐conflict region, this study found a high degree of consistency of data collection in a very difficult setting, largely owing to the presence of and reliance on local acting partners.

### Limitations

While the gold standard is believed to represent the best estimate of true events over the course of the recall period, it is important to underline that it remains an estimate: it is based on data acquired from the surveillance system and survey evaluation, both of which have biases and limitations, as described in the introduction. Although it is an imperfect benchmark for the performance of the two mortality measurement methods, it was thought to be the most accurate source for comparison given the lack of alternative records for this population.

In addition, the recall periods for the surveillance and survey do not match perfectly. While an attempt was made to mitigate this by limiting the analysis to months where both systems were complete, it is possible that some events that were included in the analysis still fell outside of this period.

It became apparent during the evaluation that some of the interviewers were attempting to differentiate between houses within (exposed) and without (unexposed) the surveillance system. In an effort to reinforce the blinding of interviewers and to reduce bias, the research team adopted a different household numbering system. Nevertheless, it was suspected for a short time afterwards that the interviewers continued to try to discern which houses were and were not under monthly surveillance. Even though all efforts were made to dissuade interviewers from discovery, there is still the potential for associated bias in estimates.

Owing to time constraints, the bulk of the evaluation data was not entered into an electronic database until after the completion of the entire study. However, the team attempted to minimise errors by instructing interviewers to find an evaluator who would check data after each individual interview. Even with the data monitoring system in place, 85 of the 1,420 households (6.0 per cent) were missing minor details associated with events (such as location of birth and reason for arrival or departure) that should have been recorded during the evaluation interview stage.

If the head of the household was not present, the team would interview another adult representative when available. It was assumed that an adult representative residing in the household would be capable of providing the necessary information. There is a chance, however, that if the representative had not spent the whole year at the house, he/she would not know about all events that occurred therein and that events would not have the same significance to a non‐head of household, making him/her less likely to recall minor events associated with short‐ and medium‐term visitors.

The large presence of local and international NGOs in Fizi Territory could also affect the reliability of the system. As a result, when an NGO visits a village, residents often believe it is to their benefit to give misleading or false information. For example, when item distributions take place within a locality, families may be signed up according to individual household or size of family. Hence, when evaluation interviewers tried to discern between households using the definition of ‘eating their meals together’, families would state that they were two or three separate households and did not eat their meals together, or include people in the household who did not eat their meals there, thinking that they would gain greater representation for the same family size if there was a distribution. This probably contributed substantially to the unusually high number of false positive births and deaths initially reported in the one‐year recall surveys.

Logistical and time constraints had a major bearing on whether or not houses were revisited when no one was home. As a rule, the team made a greater effort to revisit surveillance houses so that the evaluation and surveillance systems could be compared during analysis. The unexposed (non‐surveillance) houses were less likely to be revisited and there may have been trends particular to these houses that were missed in the evaluation.

## Conclusion

The population mobility, mortality, and birth surveillance system offers a simple and flexible means of collecting data in a timely manner, providing a stable method for high‐quality data gathering. The issue of participant fatigue needs to be addressed by locally acceptable messaging or approaches to improve the sustainability of the system. In the light of the difficulties of survey‐based evaluations with lengthy recall periods, the surveillance system appears largely to avoid data‐related pitfalls connected to household membership and recall bounds, as well as reducing respondent incentives to supply false information. Prospective surveillance can yield a detailed perspective on vital and population‐related events in the face of regional insecurity and health system duress. As such, it has the potential to endow agencies and governments working in such settings with information that is essential for making decisions about what assistance to provide, to who, and when, and ultimately, therefore, for improving the health status of populations in crisis. Prospective surveillance should be considered as an important alternative to survey techniques and should be employed to a greater degree, where possible, in humanitarian contexts.
